# Progress on the antiferromagnetic topological insulator MnBi_2_Te_4_

**DOI:** 10.1093/nsr/nwac296

**Published:** 2023-01-03

**Authors:** Shuai Li, Tianyu Liu, Chang Liu, Yayu Wang, Hai-Zhou Lu, X C Xie

**Affiliations:** Department of Physics, Harbin Institute of Technology, Harbin 150001, China; Shenzhen Institute for Quantum Science and Engineering and Department of Physics, Southern University of Science and Technology (SUSTech), Shenzhen 518055, China; Quantum Science Center of Guangdong-Hong Kong-Macao Greater Bay Area (Guangdong), Shenzhen 518045, China; Shenzhen Key Laboratory of Quantum Science and Engineering, Shenzhen 518055, China; International Quantum Academy, Shenzhen 518048, China; Shenzhen Institute for Quantum Science and Engineering and Department of Physics, Southern University of Science and Technology (SUSTech), Shenzhen 518055, China; Quantum Science Center of Guangdong-Hong Kong-Macao Greater Bay Area (Guangdong), Shenzhen 518045, China; Shenzhen Key Laboratory of Quantum Science and Engineering, Shenzhen 518055, China; International Quantum Academy, Shenzhen 518048, China; Beijing Academy of Quantum Information Sciences, Beijing 100193, China; Beijing Key Laboratory of Optoelectronic Functional Materials & Micro-Nano Devices, Department of Physics, Renmin University of China, Beijing 100872, China; State Key Laboratory of Low Dimensional Quantum Physics, Department of Physics, Tsinghua University, Beijing 100084, China; Frontier Science Center for Quantum Information, Beijing 100084, China; Hefei National Laboratory, Hefei 230088, China; Shenzhen Institute for Quantum Science and Engineering and Department of Physics, Southern University of Science and Technology (SUSTech), Shenzhen 518055, China; Quantum Science Center of Guangdong-Hong Kong-Macao Greater Bay Area (Guangdong), Shenzhen 518045, China; Shenzhen Key Laboratory of Quantum Science and Engineering, Shenzhen 518055, China; International Quantum Academy, Shenzhen 518048, China; International Center for Quantum Materials, School of Physics, Peking University, Beijing 100871, China; Institute for Nanoelectronic Devices and Quantum Computing, Fudan University, Shanghai 200433, China; Hefei National Laboratory, Hefei 230088, China

**Keywords:** MnBi_2_Te_4_, magnetic topological insulator, antiferromagnetic, quantum anomalous Hall effect, axion insulator

## Abstract

Topological materials, which feature robust surface and/or edge states, have now been a research focus in condensed matter physics. They represent a new class of materials exhibiting nontrivial topological phases, and provide a platform for exploring exotic transport phenomena, such as the quantum anomalous Hall effect and the quantum spin Hall effect. Recently, magnetic topological materials have attracted considerable interests due to the possibility to study the interplay between topological and magnetic orders. In particular, the quantum anomalous Hall and axion insulator phases can be realized in topological insulators with magnetic order. MnBi_2_Te_4_, as the first intrinsic antiferromagnetic topological insulator discovered, allows the examination of existing theoretical predictions; it has been extensively studied, and many new discoveries have been made. Here we review the progress made on MnBi_2_Te_4_ from both experimental and theoretical aspects. The bulk crystal and magnetic structures are surveyed first, followed by a review of theoretical calculations and experimental probes on the band structure and surface states, and a discussion of various exotic phases that can be realized in MnBi_2_Te_4_. The properties of MnBi_2_Te_4_ thin films and the corresponding transport studies are then reviewed, with an emphasis on the edge state transport. Possible future research directions in this field are also discussed.

## Teaser

In this review, recent advances in the intrinsic magnetic topological insulator MnBi2Te4 are reviewed from both experimental and theoretical aspects, and possible future research directions in this field are envisaged.

## INTRODUCTION

The discovery of the quantum Hall effect (QHE) opens a new chapter in condensed matter physics [1]. The quantized conductance is a manifestation of the quantum effect on the macroscopic scale; it is precisely determined in terms of fundamental constants: the electron charge *e* and the Planck constant *h*. Studies on QHE have led to a revolution in the classification of different topological phases of matter [2,3]. The concept of topological insulators (TIs) was proposed, and the corresponding materials were then found in experiments [2,3]. Subsequently, topological semimetals were theoretically predicted and experimentally realized [4,5]. In turn, these topological materials provide an ideal platform for exploring exotic transport phenomena. The quantum spin Hall effect (QSHE) and the quantum anomalous Hall effect (QAHE) were realized [6–9], both of which are supported by the distinguished edge states of topological materials in the absence of magnetic fields. Other generalizations of QHE such as three-dimensional (3D) QHE and the nonlinear Hall effect have also been explored [10]. In recent years, magnetic TIs have attracted great interests because they can host QAHE and offer opportunities to investigate the intertwined topological and magnetic orders [11]. MnBi_2_Te_4_, as the first intrinsic antiferromagnetic (AFM) topological insulator discovered, has been extensively studied.

MnBi_2_Te_4_ was first discovered and synthesized in 2013, with its thermoelectric properties investigated [12]. Riding on a wave of research on topological insulators, researchers started to pay attention to MnBi_2_Te_4_ because of its magnetism contributed by the Mn atoms. In 2017, the (MnBi_2_Te_4_ layer)-(TI film)-(MnBi_2_Te_4_ layer) heterostructure was proposed as a platform for QAHE [13]. Through first-principles calculations, the authors found a large surface magnetic gap in the MnBi_2_Te_4_ capped TI, and they concluded that the similar crystal structures and compositions of MnBi_2_Te_4_ and TI (Bi_2_Te_3_) made the out-of-plane magnetization induced by Mn more efficient. The topological properties of MnBi_2_Te_4_ were not discovered at the time. Rienks *et al.* [14] found that, instead of being a disordered system with impurities, Mn-doped Bi_2_Te_3_ developed as a heterostructure of MnBi_2_Te_4_ septuple and Bi_2_Te_3_ quintuple layers. Besides, this self-organized heterostructure held a large magnetic surface gap. With these hints, the topological properties of MnBi_2_Te_4_ were revealed theoretically by several research groups [15–18] in 2019. In the meantime, experimental studies [17,19] on the magnetic TI phase of MnBi_2_Te_4_ were conducted. Since then, studies on MnBi_2_Te_4_ started springing up [20,21].

**Figure 1.**
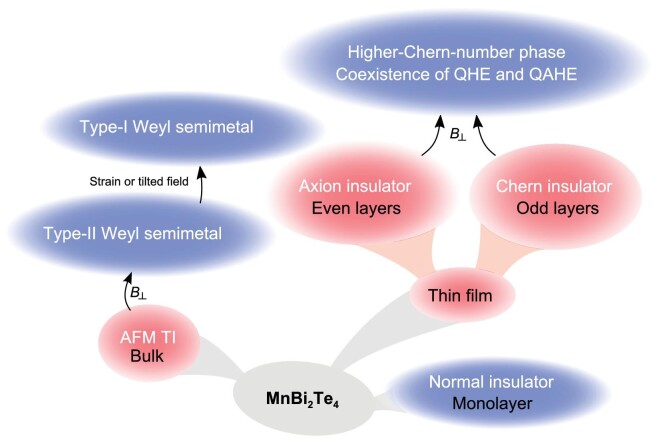
Various magnetic and topological phases in MnBi_2_Te_4_. Red denotes antiferromagnetic phases and blue denotes ferromagnetic phases.

The extensive studies on MnBi_2_Te_4_ are mainly driven by two motivations: realizing a high-temperature QAHE and exploring the exotic phases arising from its topological and magnetic orders, such as the axion insulator phase [22–24]. Before the discovery of MnBi_2_Te_4_, QAHE had been realized in magnetically doped TIs, but the extremely low temperature required by QAHE severely limited its application. In such magnetically doped TIs, magnetic moments are provided by the dopants. However, the exchange gap is very small, and thus can only survive at extremely low temperatures [25–27]. The intrinsic magnetic TI MnBi_2_Te_4_ has been expected to resolve this dilemma. On the other hand, AFM TIs were theoretically proposed in 2010 [28]. MnBi_2_Te_4_ is the first material realization of an AFM TI, providing an ideal platform to investigate the interplay between the magnetism and the topology. Figure 1 shows various exotic phases that can be realized in MnBi_2_Te_4_.

In the following, we first review the bulk MnBi_2_Te_4_, focusing on its crystal, magnetic and electronic structures, and the intensive angle-resolved photoemission spectroscopy studies on it. Then, we outline the different properties of MnBi_2_Te_4_ thin films from the bulk MnBi_2_Te_4_, and survey the studies of MnBi_2_Te_4_ films on the magnetization and the nontrivial transport phenomena, including QAHE, the zero Hall plateau and the layer Hall effect in the presence of the AFM order, and the high-temperature Chern insulator phase and the higher-Chern-number phase in the presence of the ferromagnetic (FM) order. We conclude with a discussion on the research opportunities and possible future directions.

## BULK MnBi_2_Te_4_

### Crystal and magnetic structures

**Figure 2.**
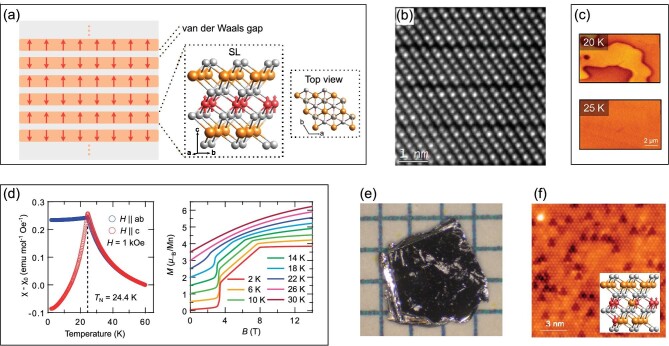
Crystal and magnetic structures of MnBi_2_Te_4_. (a) Schematic illustrations of the layered crystal structure of MnBi_2_Te_4_. Red arrows represent directions of the magnetic moments; red, yellow and gray balls represent Mn, Bi and Te atoms, respectively. (b) A scanning transmission electron microscopy image of the layered structure of MnBi_2_Te_4_. The SLs and van der Waals gaps can be clearly seen in the image. (Adapted from [29].) (c) Magnetic force microscopy images taken above and below *T*_N_ of MnBi_2_Te_4_. Domain walls emerge below *T*_N_. (Adapted from [30].) (d) Temperature-dependent magnetic susceptibility curves and field-dependent magnetization curves of MnBi_2_Te_4_. A cusp of the magnetic susceptibility curves appears at *T*_N_; the spin-flop transition happens at ∼3.5 T and the FM transition happens at ∼8 T. (Adapted from [31].) (e) A photograph of a single crystal MnBi_2_Te_4_. (Adapted from [32].) (f) Atomically resolved scanning tunneling microscopy image of the surface of a MnBi_2_Te_4_ sample. Two types of point defects are revealed: the triangular depressions and the circular protrusions. (Adapted from [33].) The inset illustrates the AFM coupling between the Mn substitution at the Bi site and Mn atoms in the Mn monoatomic layer.

MnBi_2_Te_4_ has a layered crystal structure. In the a-b plane, atoms are arranged on triangular lattices, forming monoatomic layers; in the c direction, monoatomic layers are stacked in the order Te-Bi-Te-Mn-Te-Bi-Te, constituting a septuple layer (SL; see Fig. 2(a)), and SLs are bound together by van der Waals forces (Fig. 2(a) and 2(b)). The space group of MnBi_2_Te_4_ is $R\bar{3}m$ (No. 166) [12], which is the same as the well-known Bi_2_Se_3_ family topological insulators. Compared to the quintuple layers in Bi_2_Te_3_, the SL of MnBi_2_Te_4_ has an additional Mn layer and Te layer in the middle.

The ground state of MnBi_2_Te_4_ has an AFM structure. The magnetism in MnBi_2_Te_4_ is provided by the Mn atoms. For a single SL, the magnetic moments of Mn atoms are in the ferromagnetic order pointing to the out-of-plane direction, and different SLs are coupled in the A-type AFM order along the c direction (i.e. adjacent layers have opposite magnetization directions), as sketched in Fig. 2(a). By first-principles calculations, the total energies of MnBi_2_Te_4_ with different magnetic orders [15] and magnetic exchange parameters [17] have been found, supporting the intralayer FM and interlayer AFM couplings. The energy of the A-type AFM phase with an in-plane easy axis is slightly higher than that with an out-of-plane easy axis, and the energy of the FM phase is much higher. Monte Carlo simulations have also confirmed the interlayer AFM structure with a Néel temperature *T*_N_ = 25.4 K [17]. In the experimental aspect, X-ray magnetic circular and linear dichroism experiments have been conducted [17,34]. The polarization of the Mn atoms has been verified, and an AFM order in the out-of-plane direction has been suggested. Furthermore, the same magnetic structure has also been studied in neutron diffraction experiments [32,35]. The ordered magnetic moment per Mn atom is ∼4.04 μ_B_ (where μ_B_ is the Bohr magneton) at 10 K [32]. Further inelastic neutron scattering studies [36] have shown that the AFM interlayer exchange interactions are strong, and within each Mn monoatomic layer, the next-nearest-neighbor AFM interaction *J*_2_ ≈ 0.3*J*_1_ competes with the nearest-neighbor FM interaction *J*_1_, pushing the intralayer ferromagnetism close to the classical stability limit. On the surface of MnBi_2_Te_4_, domain walls have been observed by magnetic force microscopy [30,37] (Fig. 2(c)). These domain walls disappear when the temperature is raised above *T*_N_. The width of these domain walls is relatively large (∼400 nm), and the domain size is of the order of 10 ${\mathrm{\mu m}}$.

At temperatures higher than *T*_N_, MnBi_2_Te_4_ becomes paramagnetic; under high-strength magnetic fields, it becomes ferromagnetic. Many physical properties would show discontinuity signatures at the magnetic transition. Figure 2(d) shows the temperature-dependent magnetic susceptibility curves and field-dependent magnetization curves of MnBi_2_Te_4_. The cusp of the magnetic susceptibility curves gives a Néel temperature *T*_N_ of ∼24.5 K. This feature has been widely observed in the temperature-dependent curves of magnetic susceptibility [17,31,32,34,35,38–41], magnetic torque [35], resistance [34,35,40–43], thermal conductivity [32] and specific heat [34]. When a magnetic field perpendicular to SLs is applied, the magnetic structure does not change for small field strengths; the AFM order turns into a canted-AFM order (spin-flop transition) at a magnetic field of around 3.5 T; the FM transition happens at a magnetic field of around 8 T [17,42,44].

Since some properties of MnBi_2_Te_4_ have been found to be sample dependent (especially those involving surface states), we give a brief review on the material aspect here. Bulk MnBi_2_Te_4_ crystals are usually grown from the melt of the stoichiometric mixture [45,46], via the flux method [32], or the chemical vapor transport method [40,47]. The grown MnBi_2_Te_4_ is always n-doped with a carrier concentration of ∼10^20^cm^−3^ [17,32,41–43]. By doping Sb to form Mn(Sb_x_Bi_1−x_)_2_Te_4_, the n–p carrier transition happens at *x* ≈ 0.3 [39]. Ubiquitous antisite defects have been found in the bulk MnBi_2_Te_4_ by X-ray diffraction, scanning transmission electron microscopy and scanning tunneling microscopy (STM) [32–34,40,47–49]. As shown in the STM image in Fig. 2(f), two types of point defects are revealed: the triangular depressions and the circular protrusions, where the former are Mn atoms at Bi sites and the latter are Bi atoms at Te sites. The concentration of defects is ${\sim}2\%$–4% (4%–15%) for Mn (Bi) atoms at Bi (Mn) sites, and 0.2% for the Bi/Te antisite defects [48]. In MnBi_2_Te_4_ samples grown by the chemical vapor transport method, the Mn occupancy of Mn sites and the defect Mn atoms at Bi sites are greater than that in samples grown via the flux method [40,47]. Within each SL, the Mn substitutions at Bi sites antiferromagnetically couple to the Mn atoms in the Mn monoatomic layer (see the inset in Fig. 2(f)). This is supported by classical Monte Carlo simulations and the full saturation of magnetization at ∼60 T [44]. This intralayer AFM coupling between the Mn defects and the Mn monoatomic layer further results in ferrimagnetism in each SL [33,44]. In addition to the defects in the bulk, a surface reconstruction was reported in [29], where the few-layer MnBi_2_Te_4_ measured was exfoliated from the bulk MnBi_2_Te_4_ (grown by the flux method) through a Scotch-tape method in a glovebox filled with argon. Instead of a MnBi_2_Te_4_ SL on the surface, two layers with a clear van der Waals gap were observed by high-angle annular dark-field scanning transmission electron microscopy. These two layers were identified as a Mn-doped Bi_2_Te_3_ quintuple layer and a crystalline/amorphous layer. This kind of surface reconstruction would greatly reduce the surface magnetism as the magnetic moments of Mn atoms in the crystalline/amorphous layer could be disordered.

### Electronic structure

**Figure 3.**
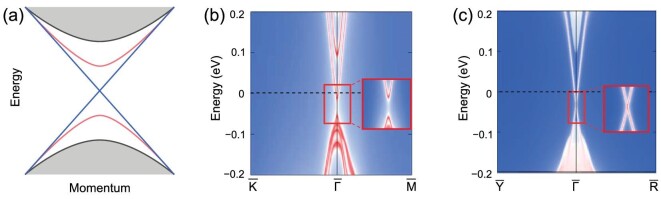
Surface states of the AFM TI MnBi_2_Te_4_. (a) Sketch of the energy bands of MnBi_2_Te_4_. Top/bottom surface state is shown in red and the side surface state is shown in blue. (b, c) First-principles calculated top and side surface states (Adapted from [16].)

Below the Néel temperature, MnBi_2_Te_4_ is an AFM TI. The AFM structure breaks the time-reversal symmetry Θ, but the spatial inversion symmetry *P* is preserved with Mn sites acting as the inversion centers. The bulk state of MnBi_2_Te_4_ has a nontrivial *Z*_2_ index protected by a combined symmetry *S* = Θ*T*_1/2_, where *T*_1/2_ is the half-unit-cell translational symmetry [15–17,28]. Besides, there exists a *P*′Θ symmetry, where the inversion center of *P*′ is located between SLs. This guarantees that each energy band is at least doubly degenerate [16,50]. Band inversion happens between the Bi $p_{z}^{+}$ band and the Te $p_{z}^{-}$ band at the Γ point, and the bulk band gap is opened by a strong spin-orbit coupling (SOC). By gradually turning on SOC in the first-principles calculations, the bulk gap first closes and then reopens, indicating a transition from a normal insulator to a TI [16,17]. Compared to the nonmagnetic TI, one of the most distinctive features of the AFM TI is the gapped surface state. In particular, in MnBi_2_Te_4_, the gapped surface states are on the top and bottom surfaces (perpendicular to the [001] direction), while the *S*-symmetry-protected side surface state remains gapless, as shown in Fig. 3. The band gaps have been calculated by first-principles calculations. The top-surface gap was predicted to be ∼90 meV inside the ∼0.2 eV bulk gap at the Γ point in [15,17]. In [16,51], the calculated energy bands showed a global gap of ∼0.16 eV at the *Z* point and a direct gap at the Γ point of ∼0.18 eV; the top-surface gap, however, was predicted to be ∼32 meV.

The low-energy effective Hamiltonian of the bulk MnBi_2_Te_4_ has been given in [15]. It is the same as that of the 3D TI [52], but with different parameters,


(1) \begin{eqnarray*}
H &=&\epsilon _{0}({\bf k})I_{4} \\
&& +\left( {\begin{array}{c@{\quad}c@{\quad}c@{\quad}c}M({\bf k}) & v_{z}k_{z} & 0 & vk_{-}\\
v_{z}k_{z} & -M(\boldsymbol{{\bf k}}) & vk_{-} & 0\\
0 & vk_{+} & M({\bf k}) & -v_{z}k_{z}\\
vk_{+} & 0 & -v_{z}k_{z} & -M({\bf k}) \end{array}}\right),\\
\end{eqnarray*}

where $\epsilon _{0}({\bf k})=C_{0}+C_{1}k_{z}^{2}+C_{2}(k_{x}^{2} +k_{y}^{2})$, $M({\bf k})= M_{0}+M_{1}k_{z}^{2}+M_{2}(k_{x}^{2}+k_{y}^{2})$, *k*_±_ = *k_x_* ± *ik_y_* and *I*_4_ is the identity matrix. The fitting parameters are *C*_0_ = −0.0048 eV, *C*_1_ = 2.7232 eVÅ^2^, *C*_2_ = 17 eVÅ^2^, *M*_0_ = −0.1165 eV, *M*_1_ = 11.9048 eVÅ^2^, *M*_2_ = 9.4048 eVÅ^2^, *v* = 3.1964 eVÅ and *v_z_* = 2.7023 eVÅ. This set of parameters only describes the energy dispersion of MnBi_2_Te_4_ near the Γ point; at a larger **k**, the particle-hole asymmetry term ε_0_(**k**)*I*_4_ bends the valence bands upwards, resulting in the disappearance of the global band gap. Another way to model MnBi_2_Te_4_ is first regularizing Equation (1) on a 3D lattice, then introducing opposite magnetization ±*H_ex_* in the neighboring layers [53],


(2) \begin{eqnarray*}
H_{ex}=\left({\begin{array}{c@{\quad}c@{\quad}c@{\quad}c}m & 0 & 0 & 0\\
0 & m & 0 & 0\\
0 & 0 & -m & 0\\
0 & 0 & 0 & -m \end{array}}\right),
\end{eqnarray*}

where *m* describes the strength of the magnetization. This model is more flexible and popular because the magnetic structure can be controlled by changing the form of *H_ex_*. In addition, an effective model for the surface states of MnBi_2_Te_4_ has been given in [54], allowing further analytical studies.

The electronic structure of MnBi_2_Te_4_ has been intensively studied by angle-resolved photoemission spectroscopy (ARPES), but its large energy gap of the top/bottom surface state remains controversial. Table 1 summarizes the non-zero $\mathrm{MnBi_{2}Te_{4}}$ top-surface gaps found in the ARPES studies.

**Figure 4.**
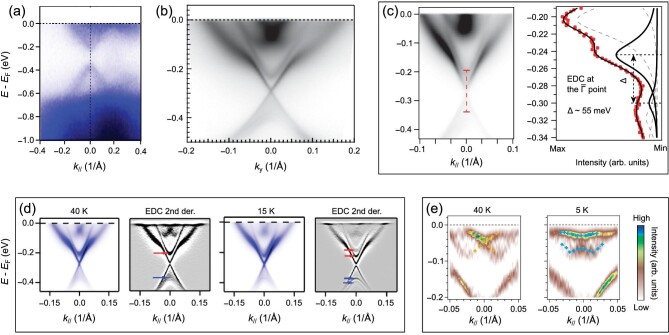
ARPES studies on the surface states of MnBi_2_Te_4_. Top panel shows the different results of the top-surface state, and the bottom panel shows other band splittings. (a) A large energy gap directly observed in the ARPES *k*-*E* map at *T* = 5 K. (Adapted from [35].) (b) Gapless top-surface state observed at *T* = 10 K. (Adapted from [31].) (c) Band gap of the top-surface state determined by peak-peak gap fitting in EDCs, measured at *T* = 10 K. (Adapted from [33].) (d) Bulk bands splitting. The valence band and conduction band are respectively marked by the blue and red arrows. (Adapted from [55].) (e) Rashba-like band splitting. (Adapted from [56].)

In the early ARPES studies, the surface gap was reported by several groups [17,35,57,58]. In [35,57], a large gap was directly found in the ARPES *k*-*E* map; see Fig. 4(a). The gap of the top-surface state was claimed to be ∼85 meV [35]. However, such an energy gap was observed at both low and high temperatures (*T* = 10 and 80 K in [57], and *T* = 5 and 300 K in [35]). This is counterintuitive because the magnetically opened gap should close when the AFM phase transitions to the paramagnetic phase at high temperatures. In [17,58], while the surface gap was not discernible in the ARPES *k*-*E* map, the authors estimated the gap size from the analysis of the energy distribution curves (EDCs) of the ARPES data; see Fig. 4(c). This means that, in the ARPES data, a finite spectral weight exists in the gap, which could be the effect of energy band broadening. A gap of ∼70 meV at *T* = 17 K was given in [17]. In [58], a gap of ∼60 meV at *T* = 10 K was revealed by fitting the peak-peak gap in EDCs, and the reduction of the gap size was roughly 15 meV when the temperature was elevated to 35 K.

In contrast to the above reports, subsequent ARPES studies reported a gapless top-surface state or a nearly vanishing gap [31,38,39,55,56,59]; see Fig. 4(b). High-resolution ARPES was used in [31,38,55,59]. For example, the laser ARPES used in [59] has an energy resolution of ∼2 meV and a momentum resolution of ∼0.005 Å^−1^. In [31], a gapless top-surface Dirac cone, existing inside the bulk band gap, was observed at both *T* = 10 and 300 K; the gapless surface state remained unchanged across the bulk Néel temperature, and was even robust against severe surface degradation; the authors suggested that a surface reconstruction of the magnetic moments might exist. In [55], from the second-derivative spectra of the EDCs at the Γ point, the surface gap was determined as 13.5 meV at *T* = 15 K and 12 meV at *T* = 40 K with an energy resolution of 4.5 meV. In addition, the band structure evolution with temperature was also studied [38]. A nearly temperature-independent gapless surface Dirac cone was observed when the temperature was changed from 7.5 to 30 K and back to 10 K; the authors suggested that the vanishing gap might be caused by multi-domains arising from different magnetization orientations [38].

Most recent studies [33,60] show that the size of the surface-state gap is strongly sample dependent, which means that defects in MnBi_2_Te_4_ crystals play an important role. From the ARPES EDCs analysis, 15 different MnBi_2_Te_4_ samples having surface gaps between 15 and 65 meV (at *T* = 10–16 K) were reported in [60]. By first-principles calculations, the authors showed that the excess surface charge could reduce the gap size. Defects in the surface region may be responsible for the excess surface charge, which further leads to different gap sizes in different samples. In [33], the defects were identified by STM and X-ray diffraction; then, surface gaps of ∼55 meV and ∼20 meV of two samples were found from the fitting of the ARPES EDCs at *T* = 10 K. By first-principles calculations, the surface state was found predominantly localized at the Bi monoatomic layers of the top-surface SL. Thus, the authors concluded that different concentrations of defects could reduce the surface gap to different degrees because the Mn defects at Bi sites were antiferromagnetically coupled to the Mn layer. Despite the fact that the observed surface gaps of different sizes can be explained by the defect effect, gap closing at temperatures above *T*_N_ has not been observed in any reports.

Although clear evidence for the surface gap opened by magnetic moments remains elusive, the magnetic effect on other energy bands has been clearly observed by ARPES in many reports [38,55,56,58,60]. Splitting of a bulk conduction band (contributed by the Te *p_z_* orbital) was observed around the Néel temperature *T*_N_ [38,55,60], and a valence-band splitting was also reported in [55]. As shown in Fig. 4(d), the valence and conduction bands, marked by the blue and red arrows, split when lowering the temperature below *T*_N_. In addition, in [31,56–58], a Rashba-like conduction band was observed at a higher energy. Since the bulk band should have the same inversion symmetry as the crystal structure, this Rashba-like band is expected to be of surface origin [56]. At the Kramers point of the Rashba-like band, the gap opening was observed when lowering the temperature below *T*_N_ [56,58], as shown in Fig. 4(e). From the analysis of EDCs, the gap size was found to be ∼30 meV at *T* = 10 K [58], and 35 meV at *T* = 5 K [56]. This Rashba-like band gap opening indicates the presence of surface ferromagnetism, but somehow it does not have a strong effect on surface states.

Infrared spectroscopy is complementary to ARPES in band structure probing [61,62]. It is a more bulk-sensitive measurement. The optical conductivity of MnBi_2_Te_4_ was found to show different temperature dependencies above and below *T*_N_ [61], and signatures of band splitting were observed [61,62]. In addition, the bulk gap was determined to be ∼0.17 eV from the interband transition [62]. These findings are consistent with the ARPES results.

In addition to the AFM TI phase, various other exotic phases can be achieved in MnBi_2_Te_4_ [15,16,51,53]. First-principles calculations showed that the bulk MnBi_2_Te_4_ with a c-direction FM order, which could be achieved via applying a magnetic field, was a type-II Weyl semimetal [15,16,51]. There is only one pair of Weyl points with a distance of ∼0.06 Å^−1^, and the Weyl points are near the Fermi energy, making MnBi_2_Te_4_ an ideal platform for Weyl semimetal studies. When the lattice constant is tuned (e.g. by strain) [15] or the direction of the magnetic moments is rotated from the out-of-plane direction to the in-plane direction [51], this type-II Weyl semimetal evolves to a type-I Weyl semimetal. In [51], MnBi_2_Te_4_ with different types of magnetic configurations was studied through first-principles calculations: when the direction of the magnetic moments was set to the in-plane direction, the two Weyl points merged together, leading to a trivial phase; MnBi_2_Te_4_ with an in-plane A-type AFM order was also predicted to be a magnetic TI, but different from the out-of-plane A-type AFM case, both top and side surfaces were found to be gapless, where the top-surface state was protected by a mirror symmetry. Furthermore, the canted-AFM phase was also studied [53], where the Möbius insulator phase and the higher-order TI phase were found.

## MnBi_2_Te_4_ THIN FILMS

**Figure 5.**
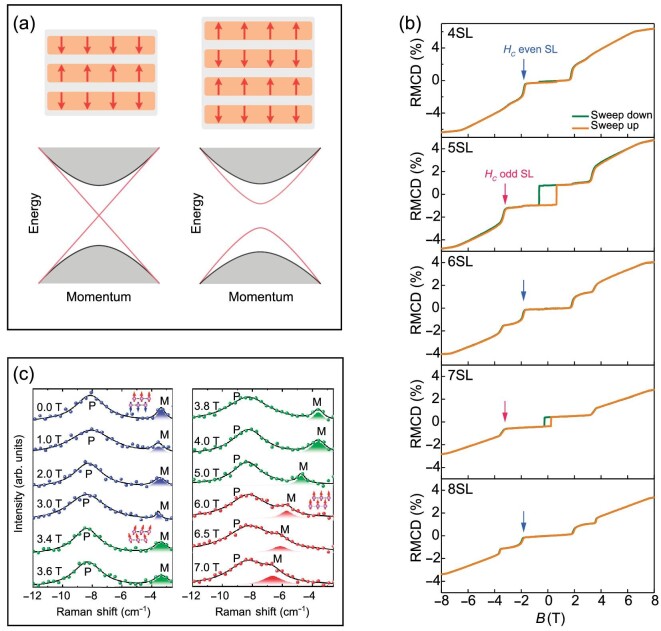
Plots of magnetic structures and energy dispersions of MnBi_2_Te_4_ films, RMCD measurements and magnon modes observed in Raman spectra. (a) Left: odd-number SLs have an uncompensated AFM magnetic structure, and a gapless edge state. Right: even-number SLs have a compensated AFM magnetic structure, and a doubly degenerate gapped edge state. (b) RMCD measurements as functions of the magnetic field at *T* = 2 K. The critical magnetic field of the spin-flop transition is marked by *H_c_*. (Adapted from [63].) (c) Raman spectra at 12 K under different magnetic fields. The colors label the magnetic orders: AFM (blue), canted AFM (green) and FM (red). Here P and M represent phonon and magnon modes, respectively. (Adapted from [64].)

MnBi_2_Te_4_ thin films can be directly exfoliated from bulk crystals or grown via molecular beam epitaxy. The thickness of each SL is ∼1.38 ± 0.4 nm, determined by atomic force microscopy [42,63,65]. In the 2D limit, MnBi_2_Te_4_ films exhibit thickness-dependent properties because of the AFM interlayer coupling. While a single SL is in the FM phase, odd-number SLs starting from three SLs have an uncompensated AFM magnetic structure, as shown in Fig. 5(a). In contrast, even-number SLs have compensated AFM structure. These magnetic structures have been confirmed by the calculations of magnetic anisotropy energies, and the total energy difference between the FM and the AFM phases [18]. The general properties of $\mathrm{MnBi_{2}Te_{4}}$ films are summarized in Table 2.

The band structure of MnBi_2_Te_4_ thin films has also been investigated by first-principles calculations [15,16,18,51,67]. The mono-SL films are normal insulators [15,16,18,67], and the band gap reported by different groups ranges from 0.3 to 0.7 eV. ARPES measurements on mono-SL films have revealed an indirect gap of ∼0.3 eV at 25 K in [19], and above 0.78 eV at 8 K in [67]. According to the band inversion analysis [51], two SL films are also trivial insulators. Found by the ARPES measurement [67], the surface band gap at 8 K is around 0.3 eV, which is much greater than those predicted by the first-principles calculations [15,16,18,51,67]. Band inversions were directly observed in three, five and seven SLs by calculating the band structure with different SOC strengths [18]. The gapless edge state in the band gap would contribute a quantum anomalous Hall conductance. For even-number SLs, the edge state is gapped [16,18]; see the sketch in Fig. 5(a). There will be a zero Hall plateau when the Fermi energy only crosses the edge state. Note that the bands are doubly degenerate in the compensated AFM even-number SLs, while the odd-number SLs break the *P*′Θ symmetry, leading to spin-split bands. As for the interior-state band gaps, both the ARPES measurements [19,67] and the tunneling conductance [49] suggest band gaps larger than those predicted by first-principles calculations [49,67].

### Studies on magnetism

The magnetic structure of MnBi_2_Te_4_ films has been studied by reflective magnetic circular dichroism (RMCD). Measurements showed that odd-number SLs had clear hysteresis loops and noticeable remanent RMCD signals in the AFM state because of the uncompensated magnetic moments, but even-number SLs had very small RMCD signals and hysteresis loops [63,68], as shown in Fig. 5(b). The small remanent RMCD signals observed in even-number SLs indicate a net magnetization, which may be caused by the substrate-induced top-bottom surface asymmetry or Mn/Bi substitution defects.

The Néel temperatures of MnBi_2_Te_4_ films are lower than that of the bulk crystal, and the temperature increases with the number of SLs before reaching *T*_N_ of the bulk crystal. The suppression of *T*_N_ is ascribed to the increased thermal fluctuations as the samples approach the 2D limit. In experiments, *T*_N_ is usually found from the kink of the temperature-dependent resistance curves. In [66], *T*_N_ of ∼20, 21, 22, 23 and 23.7 K for the three, four, five, six and seven SLs were reported, respectively. Note that *T*_N_ can be slightly different among samples [66,68–70], but its dependence on the thickness is consistent across all reports. Under the out-of-plane direction magnetic field, the critical spin-flop fields for even-number SLs are smaller than that of odd-number SLs [63,66,68], as shown in Fig. 5(b). In addition, the FM phase transition magnetic field is also smaller than that of bulk crystals [42,63,68,70].

Magnons and magnetic fluctuations were investigated using Raman spectroscopy [64], as shown in Fig. 5(c). In the AFM phase of a two-SL device, a magnon peak was found in Raman spectra with co-circularly polarized photons. This peak disappeared when the temperature was raised above *T*_N_, confirming its magnetic order dependence. Under the out-of-plane magnetic field, the observed magnon mode did not exhibit frequency dependence on the magnetic field; thus, the authors suggested that it was the two-magnon scattering that led to the observed mode. When the magnetic field turned the AFM phase to the canted-AFM and FM phases, a magnon mode with distinct field-dependent frequency shift was observed. Furthermore, by analyzing the quasi-elastic scattering, it was found that the magnetic fluctuations increased with decreasing device thickness.

### Transport phenomena in the antiferromagnetic phase

Below the spin-flop field, MnBi_2_Te_4_ films remain AFM. The different magnetization configurations of the top and bottom layers of even-number and odd-number SLs lead to different transport phenomena. Odd-number SLs were expected to host QAHE, while even-number SLs were predicted to be good candidates for axion insulators [15,16,18].

#### Quantum anomalous Hall effect

**Figure 6.**
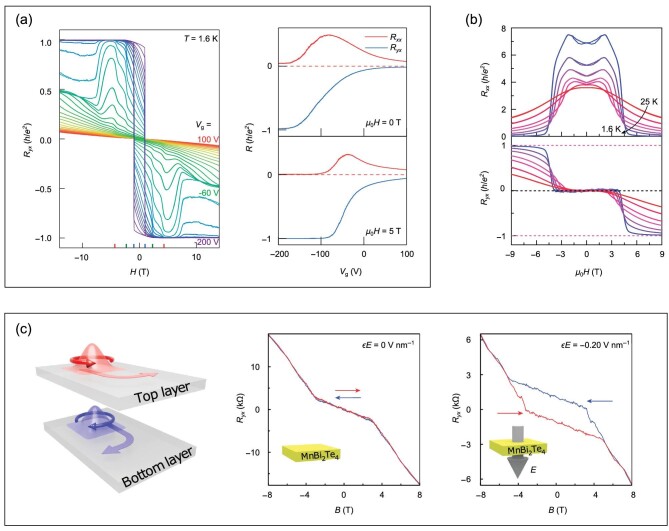
Various Hall effects in MnBi_2_Te_4_ films in the antiferromagnetic phase. (a) Quantum anomalous Hall effect in a five-SL device at *T* = 1.6 K. The left panel shows the Hall resistance as a function of magnetic field with different gate voltages, and the right panel shows the Hall and longitudinal resistances as functions of the gate voltage under different magnetic fields. (Adapted from [69].) (b) Magnetic field dependencies of the longitudinal and Hall resistances at different temperatures with a gate voltage of 25 V. A robust zero plateau appears in the Hall resistance. (Adapted from [42].) (c) Layer Hall effect observed in a six-SL device. The left panel illustrates the layer Hall effect, where electrons in the top and bottom layers are deflected in opposite directions, and the right two panels show the Hall resistances as a function of the magnetic field at zero and finite electric fields, respectively. The red (blue) curves denote the forward (backward) scan. (Adapted from [71].)

QAHE was reported in a five-SL MnBi_2_Te_4_ device at 1.4 K [69]. Figure 6(a) shows the Hall and longitudinal resistances as functions of the magnetic field and the gate voltage. With a gate voltage of −200 V, the Fermi energy of the system is close to the charge neutrality point. As shown in Fig. 6(a), at zero magnetic field, the Hall resistance *R_yx_* is 0.97 *h*/*e*^2^ with the longitudinal resistance *R_xx_* being ∼0.061 *h*/*e*^2^. By increasing the magnetic field above 2.5 T, the Hall plateau reaches 0.998 *h*/*e*^2^. Furthermore, the quantized Hall resistance was observed to stay at temperatures up to 6.5 K under a magnetic field of 7.6 T, where the zero-field Hall resistance was taken as the quantization criterion. Studies on the temperature dependence of the resistance also revealed a thermally activated behavior of the longitudinal resistance *R_xx_*; thus, a thermally activated gap could be found, which characterized the lowest energy needed to excite an electron on the valence band to the conduction band. This gap was found to be ∼0.64 meV at zero field and increased rapidly with an increasing magnetic field strength; after the magnetic field was increased to 7.6 T, the gap decreased slowly with increasing magnetic field. The evolution of the thermally activated gap can reflect the band structure variation during the AFM to FM transition. As the gap stays open, the high-field Chern insulator phase is adiabatically connected to the zero-field quantum anomalous Hall phase, i.e. they are topologically the same.

Since QAHE in the MnBi_2_Te_4_ film relies on the surface magnetic order, magnetic disorders can severely hinder the observation of QAHE. When the surface magnetism is reduced by the Mn-Bi antisite defects discussed above, the magnetic gap is reduced, and then lower temperatures are needed for QAHE. This could be the reason why QAHE in MnBi_2_Te_4_ was not widely observed.

#### Zero Hall plateau

A zero Hall plateau accompanied by a large longitudinal resistance was observed in six-SL MnBi_2_Te_4_ devices [42,72], and it was taken as the signal of realizing the axion insulator phase, which had gapped bulk and surface states and a quantized topological magnetoelectric effect [42]. In [42], transport properties of a six-SL device were investigated. When a small voltage was applied, the overall slope of the Hall traces was positive, indicating hole-type carriers; electron-type carriers dominated when a large gate voltage was applied, as evidenced by the overall negative slope of the Hall traces. However, under a moderate gate voltage, from 22 to 30 V, the Hall resistance remained zero under a magnetic field ranging from −3.5 to 3.5 T, and the corresponding longitudinal resistance reached up to 7.5 *h*/*e*^2^ (Fig. 6(b)).

Subsequently, nonlocal measurements [73] and scanning microwave impedance microscopy (MIM) [72,74] were performed to further identify the edge transport properties in the zero Hall plateau regime. In an eight-terminal six-SL device, pronounced nonlocal signals were observed, which indicated the predominance of the edge-state transport. However, the measured resistances were not quantized, showing a dissipative nature. MIM probes the local conductivity, and thus enables direct visualization of the edge state. In the experiment, MIM images were taken on a six-SL device [72]. At zero field, conductive edges were resolved; under a small magnetic field, the interior state became more insulating, and the edge state became more localized; the interior state first became conducting and then insulating upon further increasing the magnetic field (i.e. the interior gap first closed and then reopened), indicating that the system entered the Chern insulator phase. Such a feature of the interior band gap near the phase transition point was also observed in [63] by MIM and a direct bulk resistance measurement. Compared to the Chern insulator phase, the edge state in the zero Hall plateau regime is not well localized, and the corresponding interior state is not completely insulating, especially at zero magnetic field. This may imply that the surface magnetism is inhomogeneous, which can be caused by the Mn-Bi antisite defects. The observed not-well-localized edge state also explains the dissipative nature of the edge transport in nonlocal measurements.

The edge-state gap of even-number-SL MnBi_2_Te_4_ films was predicted to be a few tens of millielectronvolts in previous first-principles calculations [16]. However, recent theoretical studies tended to suggest a much smaller edge-state gap [72,75], since neither resistance nor MIM measurements detected a clear large gap signal. Furthermore, in [75], this small edge-state gap was considered to be closed by disorders. A 2D model was employed to capture the gapped-edge-state system, and the disorder was dealt by the self-consistent Born approximation. By numerically calculating the spectral function of the system in the cylindrical geometry, the edge-state gap was found to be closed at a critical value of disorder, but the topology was not changed. The transport was thus contributed by the gapless and dissipative edge states. The disorder-averaged nonlocal resistances were calculated by the Laudauer-Büttiker formalism with the disorder modeled by a random onsite potential, and they qualitatively agreed with the nonlocal measurements in [73]. The authors also suggested distinguishing this dissipative edge state by measurements with extra floating leads. Numerical calculations showed that the longitudinal conductance increased with the number of floating leads for the dissipative edge state, which is just the opposite for the nondissipative edge state in QSHE.

#### Layer Hall effect

In addition to QAHE and the zero Hall plateau, a layer Hall effect was reported in a six-SL MnBi_2_Te_4_ device [71]. This effect is analogous to the spin Hall effect and valley Hall effect. Under a magnetic field, Hall currents from the top and bottom layers have the same magnitude but flow in opposite directions, resulting in zero net Hall voltage (Fig. 6(c)). Applying an electric field in the out-of-plane direction induces an imbalance between the layers, and a large anomalous Hall conductance emerges. This layer Hall effect originates from the opposite magnetization of the top and bottom layers in even-number-SL MnBi_2_Te_4_ films. With the Fermi energy staying away from the charge neutrality point, the opposite Berry curvatures of the top and bottom layers cause carriers to be deflected in opposite directions. The application of the electric field breaks the *P*′Θ symmetry, splitting the degenerate bands. Then, the concentrations of carriers from the top and bottom layers become different, leading to a finite anomalous Hall conductance. Unlike the edge-state transport, where the magnetic disorder impedes the observation of quantization, a drop of magnetism due to the magnetic disorder only reduces the magnitude of the anomalous Hall conductance here.

In the experiment [71], dual-gated MnBi_2_Te_4_ devices were fabricated to enable simultaneous manipulation of the carrier concentration and the electric field. A large anomalous Hall effect appeared in the presence of a finite electric field, whereas no anomalous Hall effect was observed under zero electric field (Fig. 6(c)). This electric-field-induced anomalous Hall effect disappeared completely when the temperature was raised above the Néel temperature (∼21 K). When the direction of the electric field was reversed, the sign of the anomalous Hall effect flipped. Furthermore, at a fixed electric field, the anomalous Hall effect was found to show opposite signs depending on whether the system is electron doped or hole doped.

Applying an electric field to MnBi_2_Te_4_ films allows for the investigation of more rich physics. For example, the axion field was studied in [71]. As the electric field produces a different potential for each layer, the band structure can be engineered. In the AFM even-number-SL films, this band structure engineering manifests as the layer Hall effect. In a dual-gated five-SL device, sign reversal of the anomalous Hall effect was reported [65]. In the FM MnBi_2_Te_4_ films, the band structure engineering directly modifies the exchange gap, as the applied electric field acts as a switch of the Chern number [76].

### Transport phenomena in the ferromagnetic phase

Under high-strength magnetic fields, all SLs are aligned ferromagnetically. The thickness-dependent properties are smeared, for both even-number and odd-number SLs in the same FM state. A high-temperature Chern insulator phase and a higher-Chern-number phase were found in the FM MnBi_2_Te_4_ films.

#### High-temperature Chern insulator phase

**Figure 7.**
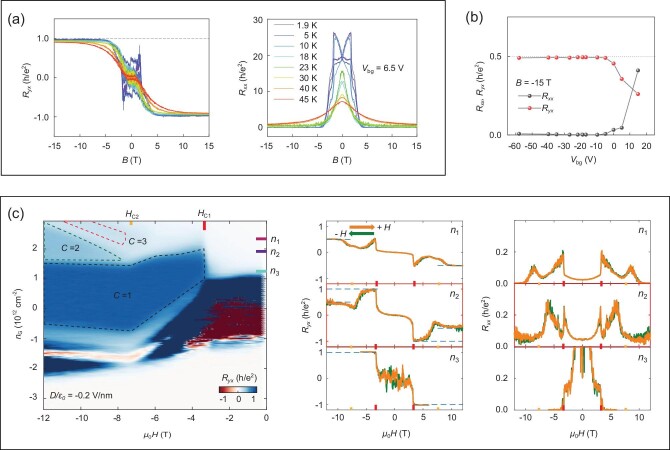
High-temperature Chern insulator phase and higher-Chern-number phase in MnBi_2_Te_4_ films in the presence of the ferromagnetic order. (a) Chern insulator phase in a seven-SL device. Under the magnetic field, the quantized conductance persists at high temperatures. (Adapted from [70].) (b) Higher-Chern-number phase in a 10-SL device. The Hall plateau of *h*/(2*e*^2^) with vanishing longitudinal resistance shows at *T* = 2 K and *B* = −15 T. (Adapted from [70].) (c) Coexistence of QHE and the Chern insulator phase in a seven-SL device at *T* = 50 mK. The left panel shows the Hall resistance as a function of carrier concentration and magnetic field, and the right panel shows the Hall and longitudinal resistances as functions of the magnetic field with different carrier concentrations *n*_1_, *n*_2_ and *n*_3_ (marked on the axis of the left panel). (Adapted from [76].)

So far, QAHE has only been reported in the above five-SL film. Most measurements on odd-number-SL devices have only given the anomalous Hall effect at zero field [65,66,69,70]. However, the quantized Hall plateau under a high-strength magnetic field was observed in many reports [40,42,63,69,70,72,74,76,77]. The combination of ferromagnetism and TI contributes an in-gap edge state, giving the *C* = −1 Chern insulator phase (Fig. 7(a)). Different from the *C* = 1 phase in the Cr-doped (Bi_1−x_Sb_x_)_2_Te_3_ for MnBi_2_Te_4_ films in the positive magnetic field, the Hall resistance reads −*h*/*e*^2^. Notably, under high-strength magnetic fields, the observed quantized Hall plateau of MnBi_2_Te_4_ films persists at high temperatures; for example, under a magnetic field above 10 T, a Hall plateau of 0.904 *h*/*e*^2^ at 45 K in a seven-SL device was reported [70] (Fig. 7(a)). As the quantized Hall plateau also arises in QHE because of the formation of Landau levels, measurements at different gate voltages were performed to distinguish this magnetic-field-induced Chern insulator phase from the traditional QHE [70]. When tuning the carriers from electron type to hole type by changing the gate voltage, the sign of the quantized Hall conductance does not change. This excludes the possibility of QHE because the occupancy of Landau levels and the sign of the Hall conductance will change once the carrier type is switched.

#### Higher-Chern-number phase

An *h*/(2*e*^2^) Hall plateau has also been observed in MnBi_2_Te_4_ films under high-strength magnetic fields [69,70,76–78]. There are two different mechanisms that can lead to this higher-Chern-number phase. In magnetic topological insulator thin films, quantum confinement can induce many sub-bands, and the higher-Chern-number phase occurs when more than one sub-band is inverted. For a given magnetic moment strength, a larger film thickness leads to a higher Chern number [79]. The second mechanism is the coexistence of QHE and the Chern insulator phase. Under high-strength magnetic fields, the interior state develops into Landau levels. When the Fermi energy crosses both the edge states contributed by QHE and the edge states contributed by the Chern insulator phase, a higher Chern number occurs.

In [70], the quantum confinement-induced *C* = −2 phase was found in a 10-SL device and a nine-SL device. Figure 7(b) shows the gate-voltage-dependent Hall and longitudinal resistances measured at *B* = −15 T in the 10-SL device. The Hall plateau is at 0.99*h*/(2*e*^2^) accompanied by a longitudinal resistance of ∼0.004*h*/(2*e*^2^). This quantized Hall resistance was reported to decrease to 0.97*h*/(2*e*^2^) when the temperature was increased to 13 K. No change in the quantized Hall resistance was observed as the dominating carriers changed from p type to n type; thus, this Hall resistance of *h*/(2*e*^2^) was contributed by two in-gap edge states induced by the quantum confinement. Further first-principles calculations showed two in-gap edge bands in the nine-SL film, and predicted a Hall resistance of *h*/(3*e*^2^) in 12-SL, 13-SL and 14-SL devices [70].

The coexistence of QHE and the Chern insulator phase was reported in [69,76–78]. As shown in Fig. 7(c), the Hall resistance develops into a plateau of −*h*/(2*e*^2^) over a certain magnetic field and gate voltage range. Unlike the above higher-Chern-number phase, this −*h*/(2*e*^2^) plateau only exists within a special gate voltage range, and there is a transition from the −*h*/*e*^2^ plateau to the −*h*/(2*e*^2^) plateau as the magnetic field increases. Theoretical studies [78] indicate that, at a small magnetic field, the interior state is localized, and only the Chern insulator edge state contributes a −*h*/*e*^2^ Hall resistance; however, at a large magnetic field, the lowest Landau level, formed by the interior state, survives the disorder, and the −*h*/(2*e*^2^) Hall plateau is caused by the coexistence of QHE and the Chern insulator phase. The phase diagram in Fig. 7(c) also shows another different characteristic between QHE and the Chern insulator phase. The carrier concentration range (i.e. the Chern insulator gap size) for *C* = 1 becomes larger when increasing the magnetic field strength, but becomes nearly fixed when |*B*| > 8 T (i.e. in the FM phase). In contrast, the *C* = 2 phase starts at a higher concentration in the FM phase, and the carrier concentration range linearly increases with the magnetic field strength. This is because the spacing between Landau levels increases with increasing magnetic field strength.

## DISCUSSION AND PERSPECTIVES

Many advances have been made in the study of MnBi_2_Te_4_, but there are still many challenges and opportunities awaiting future research. The primary concern is to synthesize high-quality samples. To keep disorders under control, more advanced crystal growth techniques are needed, and an in-depth study on the behavior of magnetic impurities is necessary. For the magnetically opened surface gap, there still lacks an understanding of the ARPES observation that the surface gap does not close at temperatures above *T*_N_. For transport studies on thin films, higher-precision device fabrication techniques are essential in order to perform layer-resolved measurements. Below, we discuss some potential research directions.

### Edge states in MnBi_2_Te_4_ even-number SLs

Realization of axion insulators has been expected since the discovery of the AFM TI MnBi_2_Te_4_, but the current results suggest that more efforts are needed. Theoretically, all surfaces of an axion insulator are gapped by magnetic orders, and the top and bottom surfaces host half-quantized Hall conductances with opposite signs. In MnBi_2_Te_4_ of even-number SLs, while the top and bottom surface gaps are opened by opposite magnetization, the side surface Dirac cone is gapped by the quantum confinement effect. A conspicuous signal of the half-quantized Hall conductance from one surface is still lacking in MnBi_2_Te_4_ thin films. Nevertheless, the enthusiasm for studying the axion insulator phase has not subsided. Here we discuss some possible future research directions in even-number-SL MnBi_2_Te_4_ films.

**Figure 8.**
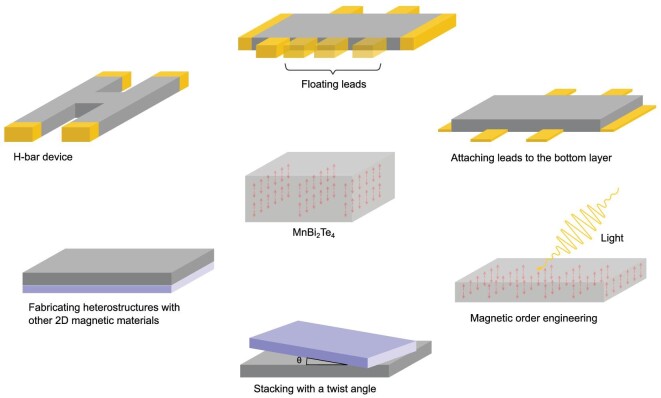
Theoretical proposals for further exploration of the edge transport and magnetic structure engineering of MnBi_2_Te_4_.

From the experimental aspect, the edge channel transport can be further explored with more different measurement methods. First, devices with different geometries can be fabricated. For example, measurements on H-bar devices (Fig. 8) allow for a more direct characterization of the nonlocal transport. Second, the relation between the resistance and the number of contacts is worth studying [75]. There are counterpropagating states at the edge of even-number SLs. Contacts can lead to an equilibration between the counterpropagating states because of the decoherence between contacts. As a result, the measured resistance is dependent on the number of contacts. However, during the measurement, adding additional contacts without affecting the sample quality can be difficult. This process can be simplified since the additional contacts added are not used. For example, additional contacts can be replaced by metallic droplets. If a metallic droplet at the edge causes full dephasing of the electronic wave function, it has the same effect as a contact [80]. Third, a direct transport measurement of the top or bottom edge is possible. To make the contacts only touch the edge of the top or bottom surface, a thicker film can be used. In this case, the side surface becomes conducting. There have been theoretical studies on the dispersion and transport when both edge and surface states exist [81,82], and the characteristics of edge states are expected to be discernible from the total transport signal. In addition to the aforementioned direct measurement of the edge transport, experiments can also be performed to detect the phase transition between the axion insulator phase and the Anderson insulator phase [83] or the unique axion magneto-optic effects [84]. Besides, experiments exploring the phase of even-number-SL films can be performed at lower temperatures or under small magnetic fields. Different from the time-reversal-symmetry protected helical edge states in QSHE, a small magnetic field does not cause a phase transition in MnBi_2_Te_4_ films, but stabilizes the edge states (as shown in MIM studies [72,74]).

From the theoretical aspect, the half-quantized Hall conductance deserves more in-depth studies. The Hall conductance of *e*^2^/(2*h*) can be directly derived from the low-energy effective surface Hamiltonian (2D gapped Dirac cone), and this value can also be found using the real-space Kubo formula in a finite lattice model [85]. However, in the Laudauer-Büttiker formalism, the physical picture of this half-quantized Hall conductance is not clear. With a voltage difference, the conductance is determined by three factors: the number of channels that support the transport, the contact conductance *e*^2^/*h* for each channel and the transmission *T*. For the *C* = 1 QAHE, there is one transport channel provided by the chiral edge state, and the transmission *T* = 1. A half-quantized conductance requires one of the aforementioned three values to be halved. Currently, the half-quantized conductance obtained by the Laudauer-Büttiker formula is based on the transmissions difference between two leads, Δ*T* = 1/2 [81,85,86]. This indicates the presence of a chiral edge current, rather than a chiral edge state; the chiral current is contributed by two counterpropagating states. The robustness of this half-quantized Hall conductance then needs to be studied. If it is robust, what protects it? When both the top and bottom surface states of an even-number-SL MnBi_2_Te_4_ film are considered, the total Hall conductance is expected to be zero. It is important to develop methods to distinguish it from QSHE [72].

### Quantum anomalous Hall effect in MnBi_2_Te_4_

QAHE is another important topic in the studies of MnBi_2_Te_4_. It is expected in odd-number-SL films below the Néel temperature, as the magnetism from the top and bottom surfaces has the same direction. So far, in MnBi_2_Te_4_ films, QAHE has only been reported in a five-SL device at 1.4 K, and there are many reports on the high-temperature Chern insulator phase under strong magnetic fields. Recently, the Chern insulator phase with a canted-AFM structure (*B* = 4 T) has been reported in a seven-SL device at 50 mK. This is a step towards the zero-field QAHE in the seven-SL device. Generally, efforts on optimizing the crystal growth are inevitable on the route to quantized transport. In previous reports, the transport properties observed varied from sample to sample; anomalous Hall signals were not found even in some odd-number-SL devices [63]. Samples of good quality can definitely produce more stable results, and are promising to realize QAHE at a higher temperature. Moreover, it is presumably more interesting and meaningful to explore a robust magnetic order or other mechanism that has an equivalent effect on the surface states. Compared to the topologically protected surface states that are robust against disorders, temperature and surface degradation, the surface magnetic orders of MnBi_2_Te_4_ can be easily broken. Finding a more reliable way to open the surface gap and induce the chiral edge state will not only advance the fundamental research but also facilitate the application of QAHE.

QAHE can also be explored in MnBi_2_Te_4_-like systems. Many studies [87–90] have shown that, in the MnBi_2_Te_4_/(Bi_2_Te_3_)_*n*_ system, increasing the number of Bi_2_Te_3_ layers in the heterostructure can gradually decrease the interlayer AFM coupling and realize an FM order. With Bi_2_Te_3_ quintuple layer termination, a gapped surface state has been observed by ARPES [88,89]. Thus, QAHE may exist in this heterostructure. Moreover, the magnetic proximity effect can be used to stabilize the FM order of the surfaces of MnBi_2_Te_4_. This can be achieved by sandwiching a MnBi_2_Te_4_ film between 2D FM insulators. Theoretical calculations have shown that CrI_3_ is a good candidate for the 2D FM insulator [91]. Compared to using the proximity effect to induce magnetization in TIs, using the proximity effect to stabilize the magnetic order in MnBi_2_Te_4_ is more promising for QAHE. Table 1, However, the induction of trivial in-gap states must be avoided in this strategy.

**Table 1.** Top-surface gap size of MnBi_2_Te_4_ found by ARPES.

Reference	Top surface gap size	Gap size determining method
Otrokov *et al.* [17]	70 meV (17 K)	Read from EDCs
Vidal *et al.* [57]	100 meV (10 and 80 K)	Read from *k*-*E* map
Lee *et al.* [35]	85 meV (5 and 300 K)	Read from *k*-*E* map
Estyunin *et al.* [58]	60 meV (10 K), 45 meV (35 K)	Peak-peak gap fitting in EDCs
Li *et al.* [55]	13.5 meV (15 K), 12 meV (40 K)	Second-derivative spectra of EDCs
Shikin *et al.* [60]	15–65 meV (10–16 K)^a^	Peak-peak gap fitting in EDCs
Garnica *et al.* [33]	55, 20 meV (10 K)^b^	Peak-peak gap fitting in EDCs

^a^Fifteen samples: for half of the samples, the gaps are in the range 28–33 meV, and a quarter of the samples have gaps between 50 and 60 meV. ^b^Two samples.

**Table 2.** Properties of MnBi_2_Te_4_ films. The band gaps are obtained by first-principles calculations [18]. The Néel temperatures *T*_N_ and the spin-flop fields can be slightly different among samples, but their dependencies on the thickness are consistent across all reports; here, the values of the Néel temperatures and the spin-flop fields are taken from [66].

	Band gap (meV)	*T*_N_ (K)	Spin-flop field (T)
1 SL	321	–	–
2 SL	107	–	2.3
3 SL	66	20	3.75
4 SL	97	21	3.5
5 SL	77	22	4.0
6 SL	87	23	3.65
7 SL	85	23.7	3.8

### Other aspects

Because of its AFM structure, MnBi_2_Te_4_ offers many opportunities in other research areas as well. As a natural AFM material, MnBi_2_Te_4_ can be used as a platform to investigate the AFM spintronics [92]. For example, surface spin-flop transitions were reported [37], and the spin filtering effect was studied [93]. The detection of hinge spin polarization in MnBi_2_Te_4_ by resistance measurement with FM contacts was proposed[94]. This can be used in the design of spin-sensitive devices. Furthermore, it is promising to explore the magnetic structure engineering of MnBi_2_Te_4_ by light. Optical tuning of the magnetic structure has been explored in other materials [95,96]; it is useful to design the desired properties.

Similar magnetic and topological properties also exist in other isostructure compounds. For example, MnSb_2_Te_4_ is often studied together with MnBi_2_Te_4_ [15,30,44,47]. Compared to MnBi_2_Te_4_, MnSb_2_Te_4_ is at the topological quantum critical point [15]; depending on the growth conditions, the prevalent Mn-Sb antisite defects lead to FM or AFM interlayer coupling [44,97]. A comprehensive study of these isostructure compounds can help find ways to optimize the magnetic and topological structures of MnBi_2_Te_4_.

By stacking MnBi_2_Te_4_ SLs with a twist angle, a moiré magnetization texture will emerge. Such texture depends on the twist angle and the number of SLs. The moiré magnetism has been reported in twisted CrI_3_ bilayers [98]. Compared to CrI_3_, MnBi_2_Te_4_ has nontrivial topological bands. A preliminary theoretical study [99] has predicted the emergence of chiral channels at the domain walls of the twisted MnBi_2_Te_4_ system and the formation of a network of such channels. Twistronics is a triumph of 2D materials in recent years [100]; therefore, further research in MnBi_2_Te_4_-based twisted systems is worthwhile.
